# Annotated dataset for deep-learning-based bacterial colony detection

**DOI:** 10.1038/s41597-023-02404-8

**Published:** 2023-07-28

**Authors:** László Makrai, Bettina Fodróczy, Sára Ágnes Nagy, Péter Czeiszing, István Csabai, Géza Szita, Norbert Solymosi

**Affiliations:** 1 Department of Microbiology and Infectious Diseases, University of Veterinary Medicine, 1143 Budapest, Hungary; 2 Centre for Bioinformatics, University of Veterinary Medicine, 1078 Budapest, Hungary; 3 Department of Physics of Complex Systems, Eötvös Loránd University, 1117 Budapest, Hungary

**Keywords:** Data publication and archiving, Bacteriology

## Abstract

Quantifying bacteria per unit mass or volume is a common task in various fields of microbiology (e.g., infectiology and food hygiene). Most bacteria can be grown on culture media. The unicellular bacteria reproduce by dividing into two cells, which increases the number of bacteria in the population. Methodologically, this can be followed by culture procedures, which mostly involve determining the number of bacterial colonies on the solid culture media that are visible to the naked eye. However, it is a time-consuming and laborious professional activity. Addressing the automation of colony counting by convolutional neural networks in our work, we have cultured 24 bacteria species of veterinary importance with different concentrations on solid media. A total of 56,865 colonies were annotated manually by bounding boxes on the 369 digital images of bacterial cultures. The published dataset will help developments that use artificial intelligence to automate the counting of bacterial colonies.

## Background & Summary

In microbiology, the colony-forming unit (CFU) is used to determine the number of viable bacteria that can grow on solid media^1^. In all cases, CFU values can only be interpreted when normalized to a unit volume (e.g., ml). In clinical microbiology, food hygiene, and vaccine research, quantification of CFU is essential. The CFU count is most commonly estimated by counting the number of colonies on solid culture media. As the estimation of the number of living bacteria is often a key, but at the same time the process of colony counting is rather time-consuming and labour-intensive, there have been several attempts in the literature to automate the procedure. A number of tools (EBImage^2^, ImageJ^3^, OpenCFU^4^, AutoCellSeg^5^, CFUCounter^6^) have been developed and are used for colony counting, which has some predefined threshold (e.g., color) and counts the resulting objects. Although they can be of great help in laboratory work, it is important to be aware of their drawbacks. A general limitation of these solutions is that objects in the image that are not colonies (e.g., pieces of the wall of a Petri dish, air bubbles) may also appear in the result as colonies. Although some tools allow these erroneous detections to be corrected manually, this again requires time-consuming expert work. Also limiting their everyday use is that most of them cannot count colonies if the number of colonies in the Petri dish is too high^6^. The use of artificial intelligence (AI) to automate colony counting seems obvious. In the AI approach, colony counting is first an object detection problem. A further task could be the differentiation of bacterial species, which requires classification solutions. By these approaches, one can obtain the total and per-class CFU count by counting the detected and classified objects to estimate the total and per-species CFU counts. There are several machine-learning approaches available to solve this kind of problem. Nowadays, convolutional neural networks (CNNs) are probably the most efficient tools in this field^7–10^, and there are efforts to use CNNs to automate colony counting^11–13^. In line with these authors, the aim of our research group was to train CNNs to estimate CFU automatically. The availability of as many digital images of annotated bacterial cultures as possible is a prerequisite for colony detection and classification with CNN. We could not find a similar public, freely available dataset to use for our own CNN-based development when we started our work.

The aim of creating the dataset presented here was to build a collection of digital records of bacterial cultures performed under everyday laboratory conditions on solid media. In creating such datasets, the question arises as to whether the digital images should be produced under some highly controlled, standardized conditions or in a way that could presumably be produced anywhere. The former solution may obviously lead to more accurate results on a given dataset, but the latter may open up the possibility of extendibility. In creating the dataset presented here and made freely available, we chose the latter approach, using mobile phones to take 369 digital images of cultures of 24 bacterial species on solid media, annotating a total of 56,865 bacterial colonies.

## Methods

### Culturing of bacterial species

Our studies have cultured 24 bacterial species of veterinary importance (Table 1). These are species whose disease processes can cause significant economic damage in farm animals, which can cause disease in companion animals, and which are important for the safety of food products. Bacterial cultures were obtained from the bacterial strain collection of the Bacteriology Laboratory, Department of Microbiology and Infectious Diseases, University of Veterinary Medicine, where the bacterial strains were stored in an ultra-low freezer at −80 °C. Before each strain is stored in the collection, its bacterial species is identified by MALDI-TOF MS. Different media were used depending on the requirements of each bacterial species (Table 1).

**Table 1:** The bacterial species included in the data set.

Bacteria	ID	Gram	Culturing	Agar	Required	
species					*NAD*	*CO*_2_
*Actinobacillus equuli*	sp01	−	aerobic	blood		
*Actinobacillus pleuropneumoniae*	sp02	−	aerobic	chocolate	+	
*Aeromonas hydrophila*	sp03	−	aerobic	blood		
*Bacillus cereus*	sp04	+	aerobic	blood		
*Bibersteinia trehalosi*	sp05	−	aerobic	blood		
*Bordetella bronchiseptica*	sp06	−	aerobic	blood		
*Brucella ovis*	sp07	−	aerobic	blood		+
*Clostridium perfringens*	sp08	+	anaerobic	blood		
*Corynebacterium pseudotuberculosis*	sp09	+	aerobic	blood		
*Erysipelothrix rhusiopathiae*	sp10	+	aerobic	blood		
*Escherichia coli*	sp11	−	aerobic	nutrient		
*Glaesserella parasuis*	sp12	−	aerobic	chocolate	+	+
*Klebsiella pneumoniae*	sp13	−	aerobic	blood, nutrient		
*Listeria monocytogenes*	sp14	+	aerobic	blood		
*Paenibacillus larvae*	sp15	+	aerobic	blood		+
*Pasteurella multocida*	sp16	−	aerobic	blood		
*Proteus mirabilis*	sp17	−	aerobic	MacConkey		
*Pseudomonas aeruginosa*	sp18	−	aerobic	blood		
*Rhodococcus equi*	sp19	+	aerobic	blood		
*Salmonella enterica*	sp20	−	aerobic	nutrient		
*Staphylococcus aureus*	sp21	+	aerobic	blood		
*Staphylococcus hyicus*	sp22	+	aerobic	blood		
*Streptococcus agalactiae*	sp23	+	aerobic	blood		
*Trueperella pyogenes*	sp24	+	aerobic	blood		

The ID column contains the unique identifier of the species, while the third column contains its Gram-staining characteristics. The culture column shows whether the bacterium requires an aerobic or anaerobic environment, and the agar column shows the medium in which it is grown. The last two columns indicate whether the species requires nicotinamide adenine dinucleotide (NAD) or *CO*_2_ during incubation.

Several steps were necessary to obtain the bacterial cultures we later used to make digital images. On the first day, the frozen strains were inoculated onto the appropriate culture medium for the bacteria and incubated under conditions appropriate to the requirements of the bacteria. On the second day, a typical colony from the culture was inoculated onto a fresh medium and incubated. On the third day, a colony of bacteria was inoculated into tryptone soy broth (TSB) using a sterile cotton swab and incubated at 37 °C for 24 hours. The cultures were then used to prepare a dilution series on a decimal basis using sterile physiological saline suspension. In the first step of the dilution (basic dilution), 0.1 ml of the initial culture was first pipetted into a test tube containing 9.9 ml sterile saline, and the suspension was thoroughly homogenized (10^−2^ dilution). Then 0.5 ml of this suspension was pipetted into a test tube containing 4.5 ml of sterile physiological saline solution. This gave the 10^−3^ dilution. The latter step of the dilution was carried out up to the 10^−6^ dilution (further dilutions).

Each member of the dilution series was homogenized by vortexing for 10 seconds. Subsequently, 50 μl per dilution of the dilutions was taken from each medium and distributed over the surface of the medium using a sterile glass rod with circular movements. After a final incubation at 37 °C for 24–48 hours, digital images of the Petri dishes containing the cultures were taken. The bacteria were inoculated, and dilutions were performed in a BSL2 safety cabinet. Incubation was done in a thermostat.

### Digitalization and annotation

For the digitalization, three different mobile phones (LG Nexus 5X, iPhone 6, and HUAWEI P30 Lite) were used so that the variability of the devices could be accounted for in the data set. For the same purpose, black and white backgrounds for the dishes were used to take the photos. Care was taken to ensure that the camera on the phone was parallel to the plane of the Petri dish.

**Fig. 1:**
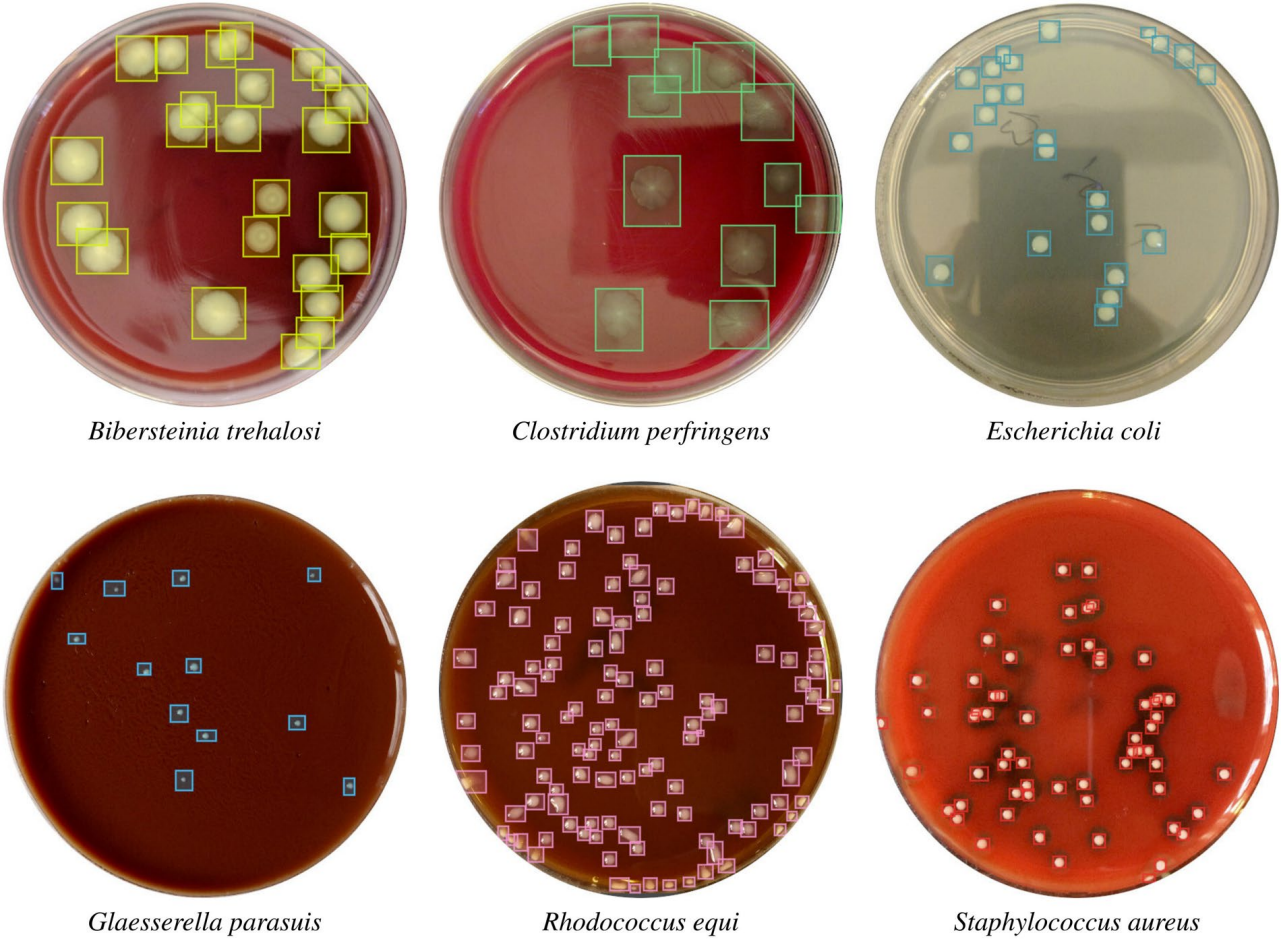
Six of 24 bacterial species cultures in Petri dishes with characteristic colonies annotated by bounding boxes. Each species has been cultured on the appropriate medium, e.g., *B. trehalosi*, *C. perfringens*, *R. equi* or *S. aureus* on blood agar, *G. parasuis* on chocolate agar and *E. coli* on nutrient agar.

The digital images were uploaded to a server where an expert using COCO Annotator v0.11.1 (https://github.com/jsbroks/coco-annotator/) drew a bounding box around each colony and labeled the identified unit with the bacterial species (Fig. 1). After annotation, the COCO^14^ structured JSON^15^ files containing the bounding boxes and labels were downloaded and subjected to further validation steps.

## Data Records

**Fig. 2:**
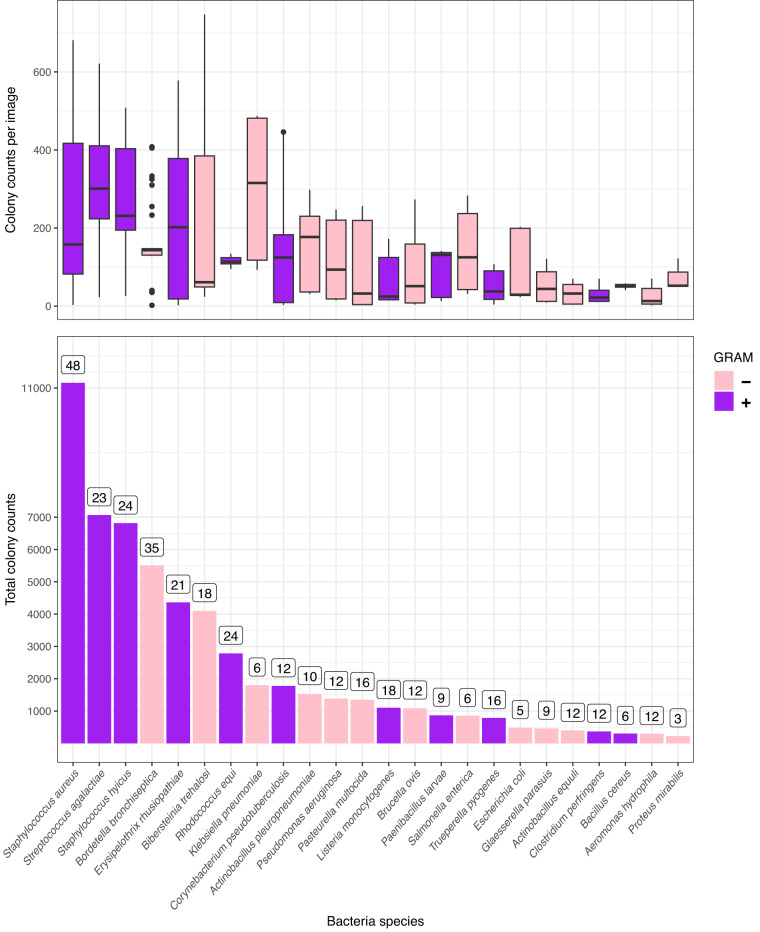
Distribution of colony counts by species and images. The barplots represent the total number of annotated colonies by species and the number of images belonging to the species in the dataset above the bars. The boxplots summarize the distribution of the annotations per image. The coloring of the graph shows the Gram-staining of the bacterial species.

The number of images, annotations per bacterial species, and the distribution of the number of annotations per image are summarized in Fig. 2. The curated 369 digital images of 24 bacterial species cultures are freely available in the Figshare repository^16^. The filenames describe their origin. The first member of the file name is the bacterial species identifier (the ID column in Table 1), and the second member is the serial number of the image associated with that species. Accordingly, the naming file sp21_img04.jpg is the 4th image of the *Staphylococcus aureus* cultures. In addition to the images, the repository^16^ contains one metadata and five annotation files. In the first sheet of the images.xls metadata file, one line for a digital image contains the bacterial species ID, the file name, whether it was taken on a white or black background, and how many CFUs it contains. All the technical characteristics of the images and their recording are listed on the second sheet. The annot_COCO.json, annot_tab.csv, annot_tab.tsv, annot_VOC_XML.zip and annot_YOLO.zip files contain the 56,865 annotation data in COCO JSON, comma-separated, tab-separated, Pascal VOC XML^17^ and YOLO^18^ formats respectively.

## Technical Validation

The annotated images of the bacterial cultures were curated by two experts with PhDs in bacteriology, and images they considered inappropriate were excluded from the final collection. The criteria for retaining images was whether the bacterial colonies morphologically matched the criteria for the species completely.

The annotations exported from the COCO annotator were reviewed by another expert using the Make Sense v1.11.0-alpha (https://github.com/jsbroks/coco-annotator/) tool, and the necessary corrections were made. In some cases, two identical images of the same culture were included in the initial collection, and these redundancies were filtered by findimagedupes v2.19.1 (https://gitlab.com/opennota/findimagedupes).

As our previous experience has shown that annotation bounding boxes exported from some annotation software can shift, especially for large numbers of annotated objects, we checked these separately. Since our CNN training designed on the dataset will be performed in the Detectron2 (https://github.com/facebookresearch/detectron2) environment, we tested whether the position of the annotation bounding boxes on the images placed with Detectron2 is correct based on the COCO format JSON files generated from the CSV files exported from Make Sense. This was done using a Python script that placed the associated bounding boxes on each digital image. The resulting images were curated one by one, and in all cases, the annotation bounding box positions were found to be correct.

Further technical validation was performed using independent data. Bärr *et al*.^19^ recorded images of *S. aureus* cultures every 10 minutes to estimate the colony growth rate. For this object detection challenge, not only the accuracy of colony detection but also the error of colony size estimation is also an important model selection criterion. We performed colony predictions on their publicly available images using CNNs trained on our dataset^10^. The colony growth rate presented by Bärr *et al*.^19^ and our predicted colony growth rate showed a difference of 0.1 *μm*/*h* (~0.2%).

## Usage Notes

As we have more experience in object detection and classification with Detectron2, we recommend this environment for using the data. As several other efficient solutions are available, we have placed the annotation data in the repository^16^ in various formats to facilitate wider use of the data.

We believe the dataset can be used for three types of object detection and classification tasks. The first option is to train neural networks to detect bacterial colonies separately per species. A second option is to treat colonies of 24 species with different morphologies as one class and train CNNs on the whole dataset to detect a “general colony-forming unit” type^10^. A third option is to train the CNN on all the bacterial culture images and annotations but using the 24 classes, allowing the classification of bacterial colonies in addition to detection.

## Data Availability

As mentioned above, the correct position of the annotations was verified by drawing the corresponding bounding boxes on the images using Detectron2. The Python script used for this is in the file bbox_placement_test.py. The input annotation file for this run is a COCO JSON one. This was also generated from the tab-delimited annotation file using a Python script provided in TSV_to_COCO.py. Both script files are available in the Figshare repository^16^.
